# Social anxiety, loneliness, and mobile phone addiction among nursing students: latent profile and moderated mediation analyses

**DOI:** 10.1186/s12912-024-02583-8

**Published:** 2024-12-18

**Authors:** Yingting Jiang, Zhenrong Shen, Yihao Zeng, Shuhan Li, Hongman Li, Ying Xiong, Zengjie Ye

**Affiliations:** 1 School of Nursing, Guangzhou University of Chinese Medicine, Guangzhou, Guangdong Province China; 2 School of Nursing, Guangzhou Medical University, Guangzhou, 510186 Guangdong Province China

**Keywords:** Nursing student, Social anxiety, Loneliness, Mobile phone addiction, Latent profile analysis, Mediation analysis, Moderation analysis

## Abstract

**Background:**

The overutilization of mobile devices by nursing students has been found to adversely affect their physical and cognitive health, potentially impeding the cultivation of a proficient nursing workforce. Previous research has identified social anxiety and loneliness as influential contributors to mobile phone addiction, but the relationship between these three factors has not been extensively examined. The objective of this research was to investigate the role of loneliness in mediating the relationship between social anxiety and mobile phone addiction, as well as the moderating influence of sleep duration on the relationship between social anxiety, loneliness, and mobile phone addiction among nursing students.

**Methods:**

We enrolled 437 students from the Be Resilient to Nursing Career program (BRNC) between October and December 2023. Surveys were employed to evaluate the levels of social anxiety, loneliness, and mobile phone addiction among nursing students. Three types of analyses were performed: latent profile analysis, mediation analysis, and moderation analysis.

**Results:**

The following three profiles of social anxiety were identified: low social anxiety (23.8%), middle social anxiety (42.8%), and high social anxiety (33.4%). The significant mediating effect of loneliness in the relationship between social anxiety based on latent profile analysis and mobile phone addiction was observed (SE = 0.709, 95%CI = 1.821, 4.618; SE = 0.561, 95%CI = 1.161, 3.345, respectively). The moderating role of sleep duration between social anxiety, loneliness, and mobile phone addiction was not significant (*P* > 0.05).

**Conclusion:**

Heterogeneity exists in social anxiety among nursing students. Loneliness serves as a significant mediating factor between social anxiety and mobile phone addiction. The moderating influence of sleep duration should be validated in future research.

## Background

By 2024, the global mobile phone user population is projected to reach an estimated 6,935.62 million [1]. Per the 53rd Statistical Report [2] on the Advancement of the Internet in China, China has the highest quantity of smartphone users globally, with 1.091 billion mobile phone Internet users as of December 2023, of which 13.7% were aged 20–29 years old. People’s life and jobs now depend a lot on their mobile phones. Nonetheless, the phenomenon of mobile phone addiction (MPA) has garnered considerable interest in research [3], as mobile devices can serve both beneficial and harmful purposes. MPA is an obsessive state of uncontrolled mobile phone usage that causes significant impairments in physical, psychological, and social functioning [4, 5]. It has been reported by a meta-analysis [6] that around 22% of nursing students (NS) exhibit symptoms of MPA. Excessive mobile phone use has various negative effects on college students: it can cause vision loss due to prolonged screen exposure [7], cervical spine injuries from poor posture [8], and a sense of detachment that diminishes the quality of social interactions and triggers negative emotions [9]. Moreover, it can impair concentration, information processing abilities, and lead to academic procrastination [10], all of which are highly detrimental to the personal development of NS.

Social anxiety manifests itself as a strong sense of fear and anxiety response in social situations out of excessive concern regarding the evaluation of others, which usually leads to individuals displaying behaviors such as avoidance and evasion in social situations [11–13]. People who experience heightened social anxiety and diminished mental well-being are at a higher risk of developing patterns of excessive mobile phone use [14]. Two meta-analyses [15, 16] have indicated a direct correlation between social anxiety and MPA.

Loneliness is commonly categorized as a subjective emotional experience or psychological awareness and a painful psychological experience when an individual’s level of interaction fails to meet expectations [13, 17]. Prior research has indicated that the likelihood of college students developing a dependency on mobile phones can be predicted by their level of loneliness, as there is a positive correlation between loneliness and MPA [18–22]. Additionally, elevated social anxiety in adolescents can result in greater challenges and setbacks in social interactions, as well as feelings of rejection and isolation from their surroundings and peers. Consequently, this can contribute to a heightened sense of loneliness and negative emotions [22]. Social anxiety is a psychological condition that serves as a direct indicator of loneliness [23].

However, there has been no empirical investigation into the possible function of loneliness as a mediator in the correlation between social anxiety and MPA [24]. Furthermore, longitudinal studies [25, 26] have demonstrated a reciprocal relationship over time between the duration of mobile phone usage and sleep duration, indicating that reduced sleep duration could significantly contribute to the onset of MPA. The overutilization of mobile devices has the potential to diminish sleep duration and result in sleep deprivation. Conversely, sleep problems can reduce inhibitory control [27], which can lead to MPA. Thus, sleep duration could potentially have a significant influence on the relationship between social anxiety, loneliness, and MPA. Therefore, we intend to explore the mediating role of loneliness in the relationship between social anxiety and MPA, and how sleep duration moderates the relationship among these three variables.

## Theoretical framework

The Interaction of Person-Affect-Cognition-Execution model (I-PACE) [28, 29] posits that person’s characteristics, affective and cognitive responses, and execution all collectively act as factors in the emergence of specific Internet use disorders. In other words, person’s characteristics (e.g., social anxiety) and affective and cognitive responses (e.g., loneliness) are vital contributors to MPA. Execution (e.g., sleep) is a cognitive and behavioral effort implemented to mitigate the adverse consequences associated with MPA. Accordingly, a moderated mediation model was developed utilizing the I-PACE model to (1) investigate the influence of social anxiety on MPA among NS, (2) analyze the mediating role of loneliness in this association, and (3) determine whether sleep duration can influence the relationship between social anxiety, loneliness, and MPA. This was done to uncover the underlying risk factors that cause MPA and to provide a conceptual framework and practical recommendations for interventions to prevent NS from developing MPA. Thus, the present research proposed the following hypotheses:

### H1

Social anxiety is a strong indicator of MPA.

### H2

Several distinct social anxiety patterns can be distinguished through latent profile analysis (LPA).

### H3

Loneliness might act as a mediating factor between LPA-based social anxiety and MPA.

### H4

Sleep duration may play a moderating role in LPA-based social anxiety, loneliness, and MPA.

**Fig. 1:**
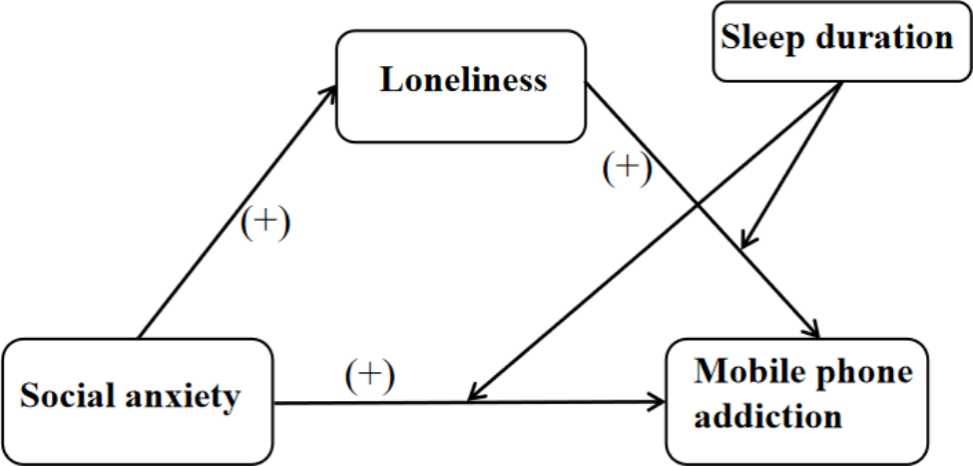
The hypothetical framework

Figure 1 details the hypothesized framework.

## Methods

### Design and participants

Between October and December 2023, NS in Guangdong Province participated in a cross-sectional study. Of the 437 NS participating in the BRNC program (a longitudinal project) [30–35], 432 individuals completed the survey, achieving a high response rate of 98.9%. The criteria for inclusion were as follows: (1) NS from freshman to senior year, (2) those who are interested in taking part in this research investigation, and (3) those who possess the ability to effectively communicate in the Chinese language. Those with mental illnesses were excluded. The participants completed paper questionnaires, requiring a time commitment of approximately 15 to 20 min. The researcher gathered and organized the questionnaires. Prior to filling out the questionnaire, consent was acquired from all participants.

### Sample size

In order to ensure the reliability and precision of subgroup outcomes, LPA requires a minimum sample size of 300 [36]. Consequently, the current study’s sample size of 432 participants demonstrated a strong statistical power.

### Instrument

#### Demographic characteristics

Demographic information included NS’ grade level, sex, birthplace, sibling status, and family finances [37].

#### Sleep duration

Based on the brief sleep duration component of the Pittsburgh Sleep Quality Index [38], NS were required to answer the first four questions of the Chinese version [39] of the scale. “Actual sleep per night” filled out by the NS was used to indicate how much sleep they were getting per night. Based on the recommendations of the Healthy China Action [40] and the National Sleep Foundation [41] on the sleep duration per night for adults, we categorized their sleep duration into short (< 7 h/night), normal (7–8 h/night), and long sleep duration (> 8 h/night).

#### Social anxiety

The Interaction Anxiousness Scale (IAS) [42] was assessed to the inclination to feel subjective social anxiety regardless of outward behavior. It demonstrated satisfactory psychometric properties in measuring the inclination to undergo personal feelings of social anxiety [43]. The Chinese version was translated by the Handbook of Mental Health Rating Scales and validated by Li et al. [44]. Fifteen items were included, with Questions 3, 6, 10, and 15 being reverse scoring questions. A Likert scale consisting of five points (1 = *not at all true to me* to 5 = *very true to me*) was used, with a total score range of 15–75. The Cronbach’s alpha coefficient derived from the present study was determined to be 0.822.

#### Loneliness

The 8-item short-form UCLA Loneliness Scale [45] adapted by Hays and DiMatteo (1987) based on the UCLA Loneliness Scale [46] was used. Zhou et al. confirmed the accuracy of the Chinese edition [47]. The scale is unidimensional and contains eight items, with the third and sixth items being inversely scored. Greater scores (rang = 0–40) on the scale suggest increased levels of loneliness. The Cronbach’s alpha coefficient obtained in this research was 0.747.

#### MPA

The MPA Index [48], a commonly used tool among Chinese college students, is utilized to assess the extent of mobile phone usage [49–51]. The Chinese version was verified by Huang Hai [52]. The scale was made up of 17 items—including “inability to control craving”, “feeling anxious and lost”, “withdrawal/escape” and “productivity loss”—in four domains. The total score ranges from 17 to 85. The scale has undergone thorough validation procedures, demonstrating strong internal consistency. This study yielded a Cronbach’s alpha of 0.894.

### Data analysis

First, demographic characteristics and sleep duration (categorical variables) were presented in terms of frequencies and proportions (%). Subsequently, a univariate analysis was utilized to investigate the possible variables that may influence the development of MPA. Second, Spearman’s correlational analysis [53] was conducted to examine the relationships among social anxiety, loneliness, MPA, and sleep duration (continuous variable). Third, utilizing the IAS scores, LPA was used to identify potential subgroups of NS with social anxiety. LPA is a method that focuses on the individual to identify latent profile classifications and examine the distinct characteristics of various segments within a population. The process commenced with a one-class model and proceeded until further enhancements of fit indices were no longer statistically significant. The subsequent fitting indices were employed to ascertain the appropriate quantity of profiles: Akaike information criteria (AIC), Bayesian information criteria (BIC), and sample-size-adjusted BIC (aBIC). Furthermore, significant Lo–Mendell–Rubin likelihood ratio test (LMR) and bootstrap likelihood ratio test (BLRT) values demonstrated that the model containing K categories outperformed the model with K-1 categories [54, 55]. Furthermore, univariate and multivariate logistic regression analyses were utilized to ascertain the factors linked with the various profiles based on LPA. The outcomes were represented through the utilization of forest plots. A Bayesian independent sample *t*-test [56] was employed to assess the differences in MPA levels across various LPA profiles. Fourth, Harman’s single-factor test was employed to assess the presence of potential common method bias [57]. Fifth, the mediating role of loneliness was estimated between LPA-based social anxiety profiles (category variable) and MPA through the PROCESS macro (Model 4). Subsequently, a moderation analysis was conducted to investigate the moderating role of sleep duration (category variable) among LPA-based social anxiety, loneliness, and MPA using PROCESS macro (Model 15) [58]. The data were run using SPSS Version 25.0 (Armonk, NY: IBM Corp), Mplus (version 8.3), and JASP (0.18.1).

## Results

### Demographic characteristics

A survey was completed by a collective of 432 NS. Participants were divided 1:4 in terms of sex. Furthermore, 49.8% of NS were from cities, and 21.8% were the only children in their families. Significant differences were identified between MPA based on sex (*P* = 0.009), annual family income (*P* = 0.048), and sleep duration (*P* < 0.001). Table 1 presents comprehensive information.

**Table 1:** Demographic and relevant variables differences in scores of mobile phone addiction

Variables	*N*	Percentage (%)	*P* value
**Gender**			0.009
Male	77	17.8%	
Female	355	82.2%	
**Grade**			0.085
Freshman	108	24.9%	
Sophomore	118	27.3%	
Junior	144	33.3%	
Senior	62	14.4%	
**Place of birth**			0.675
Cities	215	49.8%	
Countryside	217	50.2%	
**Whether the only child?**			0.911
Yes	94	21.8%	
No	338	78.2%	
**Annual family income (million yuan)**			**0.048**
<8	140	32.4%	
8–15	221	51.2%	
>15	71	16.4%	
**Sleep duration**			**< 0.001**
short	140	32.5%	
normal	281	65%	
long	11	2.5%	

Notes: Sleep duration “short”=<7 h/night, “normal”=7–8 h/night, “long”=>8 h/night

### Association between social anxiety, loneliness, MPA, and sleep duration

**Fig. 2:**
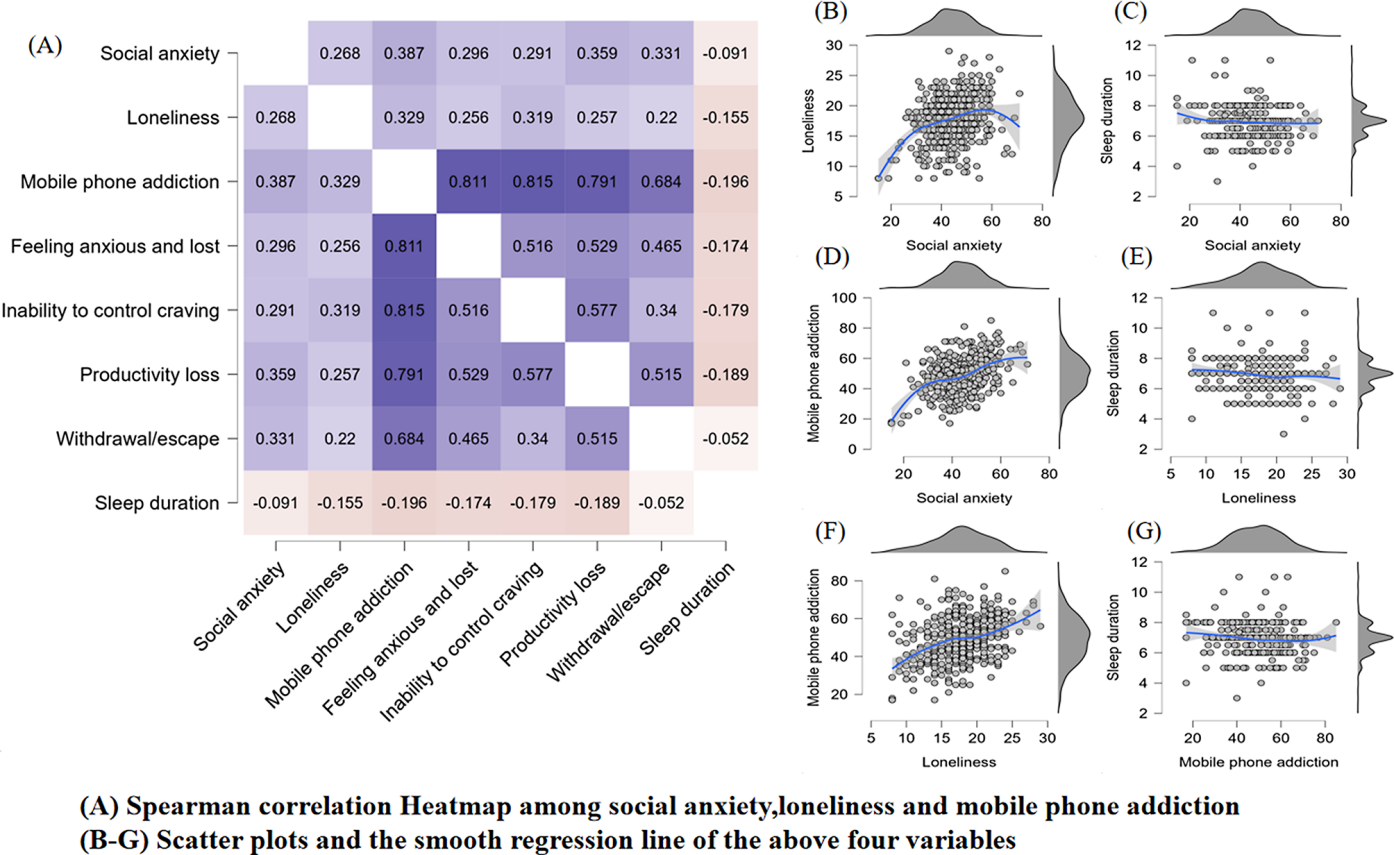
Spearman correlation Heatmap among social anxiety, loneliness, mobile phone addiction and sleep duration Notes: The bule color indicated a positive correlation, while the red color indicated a negative correlation. A darker square represents a stronger correlation

Social anxiety, loneliness, MPA, and sleep duration are all non-normal variables (Fig. 2B-G), and significant correlations were observed between them. Per Spearman’s correlation analysis, a notable positive correlation was found between social anxiety and MPA (*r* = 0.387, *P* < 0.001). Moreover, loneliness exhibited a positive association with MPA (*r* = 0.329, *P* < 0.001). Furthermore, a significant positive relationship was observed between social anxiety and loneliness (*r* = 0.268, *P* < 0.001). Figure 2A provides other details.

### LPA of social anxiety

**Fig. 3:**
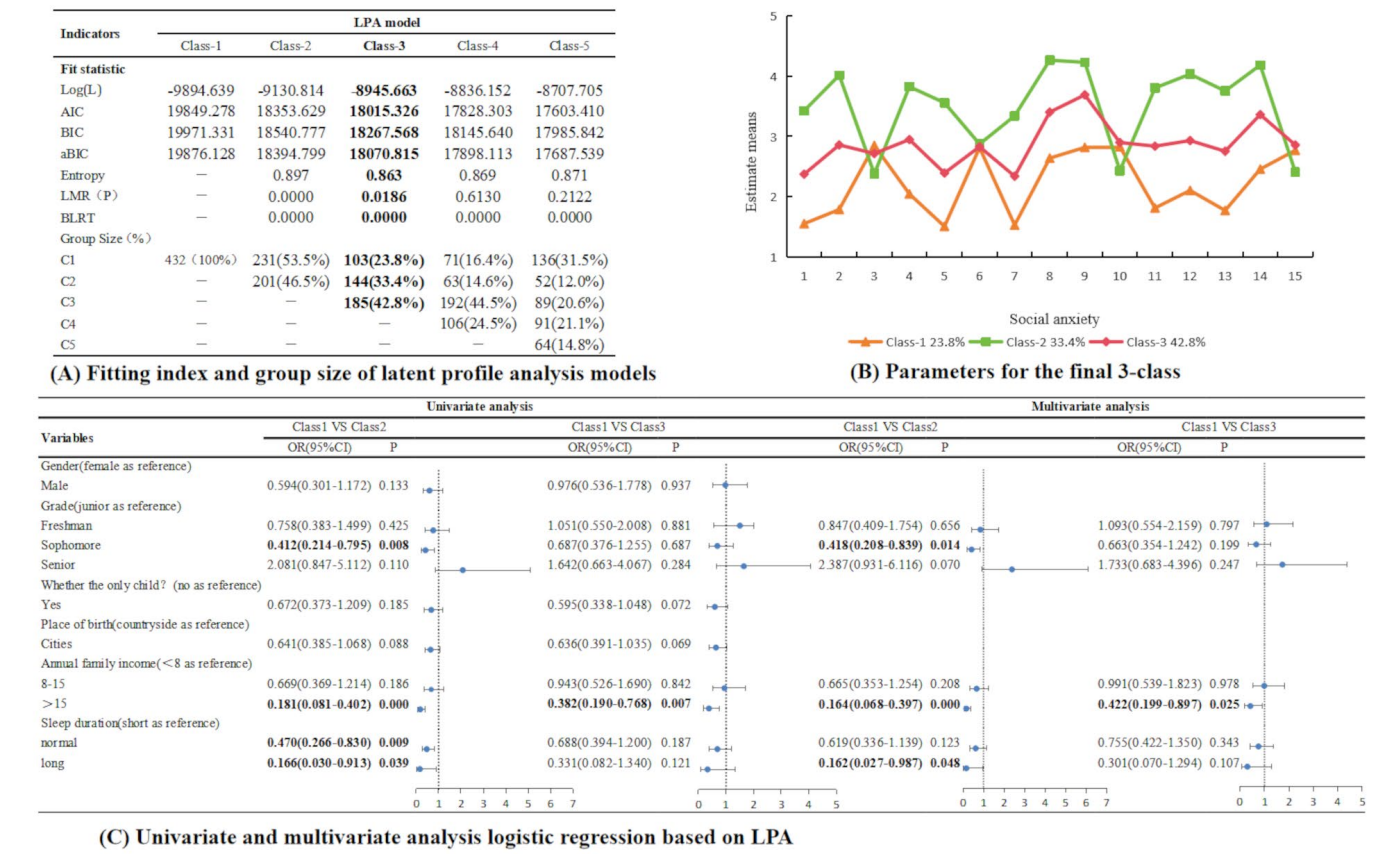
Fitting index and group size of latent profile analysis models and logistic regression results based on LPA

Figure 3A shows the fit indicators of the different LPA models. A 3-class model was chosen over a 2-class model for the following reasons: (1) The values of various fitting indices such as AIC, BIC, and aBIC were relatively low and (2) LMR and BLRT were significant (*P* < 0.05). Figure 3-B presents details about LPA-based profiles. The three profiles were termed low social anxiety (23.8%, Class1), middle social anxiety (42.8%, Class3), and high social anxiety (33.4%, Class2). Univariate and multivariate logistic regression demonstrated the importance of variables such as grade and sleep duration in differentiating between Profiles 1 and 2. Furthermore, annual family income proved to be a notable determinant of profile types. Figure 3C describes the other details.

### LPA-based difference in MPA index scale scores

**Fig. 4:**
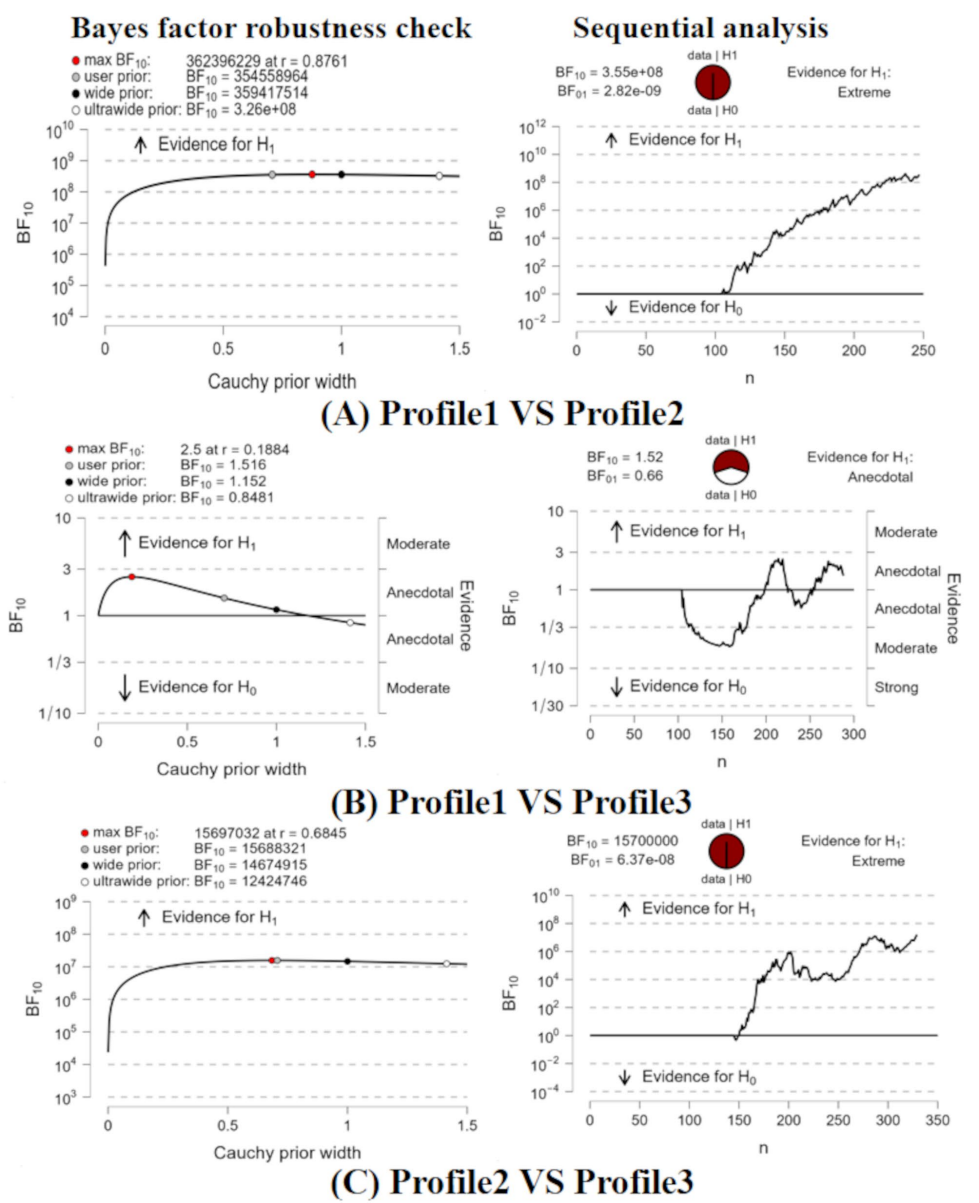
Bayes factor robustness check and sequential analysis

Substantial disparities were noted among groups categorized by varying levels of social anxiety, as evidenced by (BF10 = 3.26e + 08) for comparisons between low and high social anxiety groups, (BF10 = 0.8481) for comparisons between low and middle social anxiety groups, and (BF10 = 12424746) for comparisons between high and middle social anxiety groups. Figure 4 presents additional and detailed information.

### Mediating role of loneliness based on LPA

Harman’s single-factor model demonstrated that the initial factor explained 24.5% of the overall variances, suggesting that the potential influence of common method bias was deemed negligible for the purposes of this study. Before starting the analysis, we ensured that all potential confounding variables were controlled and low social anxiety (Class-1) was used as the reference. In Model 1 (Class-2 vs. Class-1), the 95% confidence intervals were as follows: indirect effect (1.849,4.530), direct effect (3.908,9.823), and total effect (7.026,12.828). The findings indicated that loneliness served as a significant mediator in the relationship between high social anxiety and MPA. In Model 2 (Class-3 vs. Class-1), the results showed the following findings: indirect effect (1.162,3.315), direct effect (-1.985,3.398), and total effect (0.126,5.543). These results suggest that loneliness served as a significant mediating factor, demonstrating a full mediation effect between middle social anxiety and MPA. Table 2 describes the other details.

**Table 2:** The mediation effect of loneliness on LPA-based social anxiety

Variables	β	SE	t	*P*	LLCI	ULCI	*R* ^2^
	Outcome variable: loneliness (low social anxiety as reference)						0.116
High social anxiety	3.611	0.491	7.354	< 0.001	2.646	4.576	
Middle social anxiety	2.510	0.458	5.475	< 0.001	1.609	3.411	
	Outcome variable: mobile phone addiction (low social anxiety as reference)						0.206
High social anxiety	6.866	1.505	4.562	< 0.001	3.908	9.823	
Middle social anxiety	0.706	1.369	0.516	0.606	-1.985	3.398	
Loneliness	0.848	0.140	6.067	< 0.001	0.573	1.122	
	Direct and indirect effect of loneliness on mobile phone addiction (low social anxiety as reference)						
	Variables	Effect	SE	t	LLCI	ULCI	
Indirect effect	High social anxiety	3.061	0.682	—	1.849	4.530	
	Middle social anxiety	2.128	0.556	—	1.162	3.315	
Direct effect	High social anxiety	6.866	1.505	4.562	3.908	9.823	
	Middle social anxiety	0.706	1.369	0.516	-1.985	3.398	
Total direct	High social anxiety	9.927	1.476	6.726	7.026	12.828	
	Middle social anxiety	2.834	1.378	2.057	0.126	5.543	

### Moderating role of sleep duration (category variable) between loneliness and MPA

**Fig. 5:**
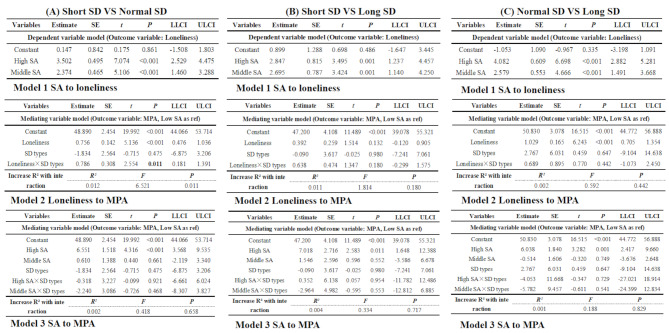
The moderation effect of sleep duration between social anxiety, loneliness and mobile phone addiction Abbreviations: “SA” = “social anxiety”, “SD” = “sleep duration”, “MPA” = “mobile phone addiction”

Potential confounding variables such as grade and annual family income were taken into account for prior to the study. The moderation analyses (Fig. 5A Model 2) indicated that the relationships between loneliness and different types of sleep duration (short sleep duration and normal sleep duration) were linked to MPA (*B* = 0.786, SE = 0.308, *P* = 0.011). Nevertheless, there was no notable moderating effect observed between groups with short sleep duration and long sleep duration, and between groups with normal sleep duration and long sleep duration (*B* = 0.638, SE = 0.474, *P* = 0.180; *B* = 0.689, SE = 0.895, *P* = 0.442, respectively). Figure 5A-C (Model 2) presents other information.

### Moderating role of sleep duration (category variable) between LPA-based social anxiety (category variable) and MPA

The group with low social anxiety was used as the reference, and all possible confounding factors were precluded through control measures. The moderating role of sleep duration types between LPA-based social anxiety and MPA was not significant (R^2^ = 0.002, *F* = 0.418, *P* = 0.658; R^2^ = 0.004, *F* = 0.334, *P* = 0.717; and R^2^ = 0.001, *F* = 0.188, *P* = 0.829, respectively). Figure 5A-C (Model 3) describes the other details.

## Discussion

Heterogeneity was observed in NS’ social anxiety. Loneliness could mediate the relationships between social anxiety and MPA. Furthermore, sleep duration did not act as a moderating factor in the associations among social anxiety, loneliness and MPA.

First, there was a positive correlation observed between social anxiety and MPA, consistent with findings from prior research [59, 60]. In a meta-analysis [15], it was found that social anxiety served as a notable predictor of MPA among adults. Socially anxious individuals are nervous and fearful when interacting with others, especially strangers; and they are very apprehensive and unconfident about presenting themselves in public social situations. The cognitive-behavioral model [61] indicates that socially anxious individuals perceive others as critical and believe they may be judged negatively, which leads to avoidance behaviors in interpersonal situations. Whereas the discomfort of realistic interpersonal interactions can be avoided to some extent by using a mobile phone, people exhibit avoidance of interruptions by viewing short, interesting videos on the screen or pretending to type in order to appear to be busy. Individuals with social anxiety disorder perceive a reduced probability of being socially threatened in online interactions [62]. Therefore, when an individual experiences social anxiety, online communication via a mobile phone can be a safer and more comfortable alternative to real-life socialization, particularly through text messaging and social media interactions. However, this may contribute to an increased likelihood of developing an addiction to mobile phone use [62–64]. Congruent with H2, heterogeneity of social anxiety was verified in NS, and the following three profiles were identified via LPA: low, middle, and high. In addition, LPA demonstrated that students with short sleep duration (< 7 h/night) were prone to high social anxiety, aligning with prior research on the subject [65, 66].

Second, consistent with H3, the mediating factor of loneliness between social anxiety and MPA was confirmed. In the first part of the mediation pathway, a positive correlation was observed between social anxiety and loneliness, aligning with findings from prior research [23, 67]. When individuals are participating in social activities, they subjectively predict that people around them may evaluate their speech, behavior, and other aspects negatively, which will produce fear and anxiety. This is the expression of the fear of negative evaluation [68], and it also produces the experience of social anxiety [69]. Several studies have concluded that the fear of negative evaluation serves as a core characteristic of social anxiety [70, 71] and is an important risk factor for inducing social anxiety in individuals [72, 73]. This fear tends to produce an inferiority complex and a defensive mindset, leading to social avoidance among college students [74]. In turn, this avoidance affects their normal social interactions and creates a sense of loneliness [75, 76]. The second pathway of the mediating mechanism between loneliness and MPA, as revealed in the present study, aligns with prior research that demonstrate a positive correlation between loneliness and MPA [18–21]. It proves that when individuals feel out of place in the physical world, they tend to resort to mobile phones (virtual networks) to escape negative emotions such as loneliness [77]. Universities can reduce college students’ loneliness by strengthening interpersonal education, providing psychological counseling services, encouraging participation in club activities, and cultivating students’ hobbies and interests [78], thereby preventing MPA.

Third, sleep duration did not significantly moderate among LPA-based social anxiety, loneliness, and MPA. There is a significant result showing that as loneliness increases, the normal sleep duration group increases MPA relative to the short sleep duration group, which is contrary to previous research [79, 80]. Studies have shown that college students generally stay up late, and their average sleep time is around 7 h [81], resulting in only 2.5% of the population in the long-sleep group (> 8 h/night) being collected in this study, and a relatively small percentage of the population in the short-sleep group (< 7 h/night). The uneven distribution of the samples of sleep duration leads to biased parameter estimations in the regressions, and these findings require further validation in future research.

### Limitations

The current research exhibits various constraints. First, the sample collected is derived from NS in Guangdong Province, which might not be representative. Thus, the findings of the present research may not be applicable to NS in other regions. Second, this cross-sectional study fails to explore their causal relationship. Hence, the sample should be expanded with longitudinal studies to further validate the correlations in the present study. Third, sleep duration was collected using scale questions, which may have been subject to recall bias. Future studies could use sophisticated sleep monitoring instruments. Furthermore, the distribution of sleep duration was not equalized. Future studies will have to adopt a stratified sampling method based on expanding the sample to ensure that individuals with different sleep durations are appropriately represented in the sample.

## Conclusion

Heterogeneity exists in social anxiety among NS. Loneliness serves as a significant mediating factor between social anxiety and MPA. Additionally, sleep duration cannot significantly moderate the associations between social anxiety, loneliness, and MPA, which should be validated further.

## Data Availability

The data that support the findings of this study are available from the corresponding author upon reasonable request.
